# Flavonoids as a Natural Treatment Against *Entamoeba histolytica*

**DOI:** 10.3389/fcimb.2018.00209

**Published:** 2018-06-22

**Authors:** Moisés Martínez-Castillo, Judith Pacheco-Yepez, Nadia Flores-Huerta, Paula Guzmán-Téllez, Rosa A. Jarillo-Luna, Luz M. Cárdenas-Jaramillo, Rafael Campos-Rodríguez, Mineko Shibayama

**Affiliations:** ^1^Departamento de Infectómica y Patogénesis Molecular, Centro de Investigación y de Estudios Avanzados del Instituto Politécnico Nacional, Ciudad de México, Mexico; ^2^Sección de Estudios de Posgrado e Investigación, Instituto Politécnico Nacional, Escuela Superior de Medicina, Ciudad de México, Mexico; ^3^Coordinación de Morfología, Departamento de Formación Básica Disciplinaria, Instituto Politécnico Nacional, Escuela Superior de Medicina, Ciudad de México, Mexico

**Keywords:** *Entamoeba histolytica*, flavonoids, alternative treatment, anti-oxidants, anti-inflammatory response, metronidazole

## Abstract

Over the past 20 years, gastrointestinal infections in developing countries have been a serious health problem and are the second leading cause of morbidity among all age groups. Among pathogenic protozoans that cause diarrheal disease, the parasite *Entamoeba histolytica* produces amebic colitis as well as the most frequent extra-intestinal lesion, an amebic liver abscess (ALA). Usually, intestinal amebiasis and ALA are treated with synthetic chemical compounds (iodoquinol, paromomycin, diloxanide furoate, and nitroimidazoles). Metronidazole is the most common treatment for amebiasis. Although the efficacy of nitroimidazoles in killing amebas is known, the potential resistance of *E. histolytica* to this treatment is a concern. In addition, controversial studies have reported that metronidazole could induce mutagenic effects and cerebral toxicity. Therefore, natural and safe alternative drugs against this parasite are needed. Flavonoids are natural polyphenolic compounds. Flavonoids depend on malonyl-CoA and phenylalanine to be synthesized. Several flavonoids have anti-oxidant and anti-microbial properties. Since the 1990s, several works have focused on the identification and purification of different flavonoids with amebicidal effects, such as, -(-)epicatechin, kaempferol, and quercetin. In this review, we investigated the effects of flavonoids that have potential amebicidal activity and that can be used as complementary and/or specific therapeutic strategies against *E. histolytica* trophozoites. Interestingly, it was found that these natural compounds can induce morphological changes in the amebas, such as chromatin condensation and cytoskeletal protein re-organization, as well as the upregulation and downregulation of fructose-1,6-bisphosphate aldolase, glyceraldehyde-phosphate dehydrogenase, and pyruvate:ferredoxin oxidoreductase (enzymes of the glycolytic pathway). Although the specific molecular targets, bioavailability, route of administration, and doses of some of these natural compounds need to be determined, flavonoids represent a very promising and innocuous strategy that should be considered for use against *E. histolytica* in the era of microbial drug resistance.

## Introduction

*Entamoeba histolytica* is a protozoan pathogen in humans and is the causative agent of amebiasis. This disease produces 100,000 deaths each year, particularly in developing countries. *E. histolytica* can manifest as a commensal or invasive microorganism in the large intestine, producing amebic colitis. The parasite has two cell cycle stages, the cyst and the trophozoite. The cysts are secreted in the stool from individuals who harbor *E. histolytica*. The trophozoites emerge from the cysts in the intestine, and this vegetative form is able to invade the intestinal mucosa and to disseminate to the liver, producing amebic liver abscess (ALA) (Sehgal et al., 1996; Haque et al., 2003). Few infected persons show symptoms with invasion to the bowel or extraintestinal sites, mainly in the liver (Stanley, 2003).

Diverse drugs have been employed against amebiasis. The treatment depends on the diagnosis and severity of the illness (Table 1). Usually, the drug can attack luminal forms and invasive amebiasis. However, in symptomatic patients and in invasive disease, the most widely used drugs against *E. histolytica* are the nitroimidazoles (metronidazole and tinidazole) (Marie and Petri, 2013; Ansari et al., 2015). Metronidazole (MTZ) kills amebas but does not cause damage to cysts. Although MTZ is the standard compound for treating amebiasis, it causes adverse effects, such as diarrhea, metallic flavor, loss of appetite, and nausea due to the doses and long-term treatment (1.5 g/day for 10 days) (Haque et al., 1997, 2003; Stanley, 2003). There are *in vivo* and *in vitro* studies where the nitroimidazoles induce genotoxic effects, which are related to the ability of cells to reduce these drugs; moreover, the position of the CH_3_ and NO_2_ groups of these compounds are involved in DNA damage (Boechat et al., 2015). In the case of MTZ, it is known that its biotransformation produces nitroso intermediates (e.g., hydroxy metabolite and acetic acid), which can form adducts in the DNA or inhibit the thioredoxin reductase-generating reactive oxygen species, causing oxidative cell damage (Mudry et al., 1994; Elizondo et al., 1996; Leitsch et al., 2007). Furthermore, MTZ can cross the blood-brain barrier producing cerebellar toxicity (Agarwal et al., 2016). Thus, it is important to consider the doses of MTZ administration, the individual susceptibility and to evaluate the risk-benefit in relation with the severity of the infection (Mudry et al., 1994; Elizondo et al., 1996).

**Table 1:** Pharmacological treatment of amebiasis.

**Amebiasis**	**Drug**	**Doses**	**Advantages**	**Side effects**	**References**
Asymptomatic carrier	Teclozan	500 mg orally (two times a day) over 3 days	High efficacy (80 and 93%) Few side effects No teratogenic effect	Abdominal pain, flatulence, and nausea	Werner Apt, 2014
	Etofamide	500 mg orally (three times a day) over 3 days	Good efficacy (92%) Long half life Short treatment	Flatulence, vomiting, urticaria, pruritis	Werner Apt, 2014
	Diloxanide furoate	500 mg orally (three times a day)	High efficacy (83%) Rapidly absorbed Low toxicity	Flatulence, nausea, and vomiting	Pehrson and Bengtsson, 1983
	Paromomycin	30 mg/kg/day	High efficacy (85%) Use during breastfeeding No nephrotoxicity	Diarrhea, gastrointestinal symptoms	Haque et al., 2003
Amebic colitis	Metronidazole	750 mg orally (three times a day) over 7–10 days	High efficacy (90%) Good bioavailability	Anorexia, nausea, diarrhea, and metallic aftertaste	Li and Stanley, 1996; Haque et al., 2003; Marie and Petri, 2013
	Tinidazole	800 mg orally (three times a day) over 7 days	Long half life Good distribution in tissues	Anorexia, nausea, diarrhea, vomiting, and metallic aftertaste	Pehrson and Bengtsson, 1983
	Iodoquinol	650 mg orally (three times a day)	Highly selective	Headache, nausea, vomiting, and optic nerve damage	Stanley, 2003
Amebic liver abscess (ALA)	Metronidazole	750 mg IV over 5–10 days	High efficacy (90–100%) good bioavailability in tissues	Dizziness, headache, gastritis, diarrhea, nausea, vomiting, stomach upset, and metallic taste	Calleja Bello and Colin Abarranco, 1979; Li and Stanley, 1996; Haque et al., 2003; Marie and Petri, 2013
	Tinidazole	800 mg orally (three times a day) over 7 days	Long half life Good distribution in tissues	Anorexia, nausea, diarrhea, vomiting, and metallic aftertaste	Pehrson and Bengtsson, 1983

Because of the undesirable effects of MTZ, it is necessary to develop an alternative treatment from biological or synthetic sources that can eliminate *E. histolytica*. Specifically, the development or discovery of novel compounds without toxicity and side effects is needed. In many countries, plant extracts have been employed ancestrally and are a good alternative to treat amebiasis. Furthermore, it is necessary to validate, identify and purify the active compounds with anti-amebic properties before employing them as a treatment.

Usually, in plants, polyphenolic metabolites can be present in roots, leaves, flowers, and fruits. Flavonoids are contained within these natural compounds, which have demonstrated promising results mainly in *in vitro* studies; however, it is necessary to evaluate their potential activity in *in vivo* models as a complementary and/or specific therapeutic strategy against *E. histolytica* trophozoites, as we describe below.

## Flavonoid structure and classification

**Figure 1:**
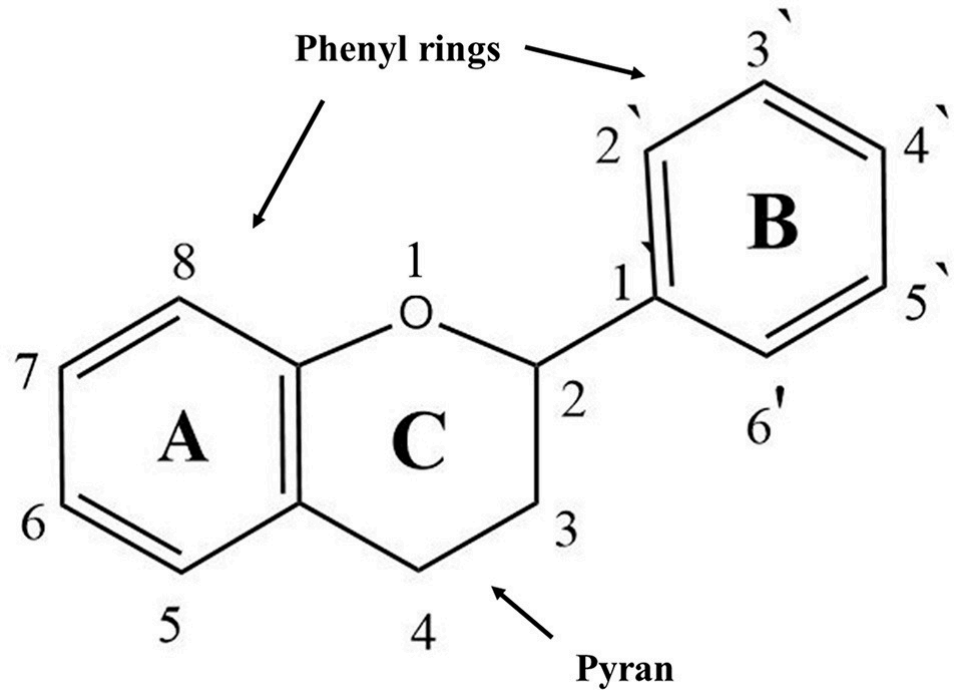
General structure of flavonoids.

Flavonoids are natural pigments present in vegetables. These compounds protect organisms against the damage produced by oxidant agents such as UV radiation, pollution, and the chemical substances present in the foods. Humans obtain these protector flavonoids through direct ingestion of aliments and supplements. The chemical structure of flavonoids is characterized by the presence of a variable number of phenolic hydroxyl groups (polyphenols). Flavonoids have low molecular weights (500–4,000 Da) and share a common skeleton of diphenylpyranes (C6-C3-C6) composed of two phenyl rings linked through a ring C of pyran (Figure 1). These compounds present excellent iron-chelating ability that gives them great anti-oxidant capacity (Havsteen, 1983). The anti-oxidant activity of flavonoids depends on the redox properties of their phenolic groups (Bors et al., 1990). According to their structural characteristics (the nature of the C3 element), flavonoids can be classified in Table 2. Different chemical modifications may occur in each group, such as hydroxylation, hydrogenation, sulfuration, methylation, acylation, and glycosylation (Andersen and Markham, 2006; Wang et al., 2017).

**Table 2:** Classification of flavonoids according to structural arrangement.

**Classification**	**Structural characteristic**	**Examples**	**References**
Flavones	They have a double bond between position 2 and 3 and a ketone in position 4 of the C ring	Apigenin, luteolin, acacetin, chrysin	Panche et al., 2016
Flavonols	They have a –OH group in position 3 of the C ring, which may also be glycosylated	Quercetin, kaempferol, rutin, and myricetin	Panche et al., 2016
Flavanones	Flavanones also called dihydroflavones, have the C ring saturated	Hesperetin, naringenin	Grayer and Veitch, 2006; Kumar and Pandey, 2013
Flavan-3-ols (Flavanols)	Has an -OH group in position 3 of the C ring	(+)-cathechin, (–)-epicatechin, (–)-epigallocatechin, (–)-epicatechingallate, (–)-epigallocathechingallate, and (+)-gallocatechin	Naczk and Shahidi, 2004; Panche et al., 2016
Anthocyanidins (Anthocyanins)	Has the -OH group in position 3, but also have a double bond between carbons 3 and 4 of C ring	Delphinidin, cyanidin, pelargonidin, petunidin, peonidin, and malvidin	Middleton et al., 2000; Nijveldt et al., 2001
Chalcones	Chalcones form a wide range of dimers and oligomers, they are characterized by the absence of C ring of the basic skeleton structure	Butein, okanin, phloridzin, arbutin, phloretin, and chalconaringenin	Naczk and Shahidi, 2004
Isoflavonoids	Often referred as phytoestrogens, have the B ring in position 3 of the pyran	Genistein, genistin, daidzein, daidzin, biochanin A, formononetin	Pietta, 2000

### Flavones

The flavone family is a subgroup of flavonoids that is synthesized depending on whether they contain *C*- or *O*-glycosylation and a hydroxylated B-ring. These compounds have been mainly isolated from leaves, aerial parts, and the exudates of plants. Flavones are characterized by a double bond between C-2 and C-3 and a B ring in C-2 (Jiang et al., 2016). Flavones present anti-oxidant activities due to their ability to scavenge reactive oxygen species (ROS). For example, luteolin inhibits xanthine oxidase, which is an important enzyme that is involved in ROS production (Cos et al., 1998; Spanou et al., 2012). Apigenin reduces the phosphorylation of NF-κB/p65 in mouse macrophages and in human monocytes, inhibiting its transcriptional activity and the expression of pro-inflammatory cytokines (Nicholas et al., 2007). Acacetin has shown important anti-peroxidative and anti-inflammatory activities, and these effects were determined by inhibiting iNOS and COX-2 activity in murine macrophages (Pan et al., 2006) (Table 2).

### Flavonols

Flavonols are composed of multiple phenol structural units. Examples of this group are kaempferol, kaempferol-3-methyl ether, quercetin, and quercetin-3-methyl ether among others. The flavonols present important health benefits, such as the anti-oxidant activity, by increasing the activity of catalase and glutathione peroxidase (Bai et al., 2016). They also act as radical scavengers (Tipoe et al., 2007; Bai et al., 2016). In addition, they present anti-inflammatory effects by inhibiting the activity of lipoxygenase and cyclooxygenase (Kim et al., 1998; Marunaka, 2017).

### Flavanones

This group is characterized by the lack of a double bond between C-2 and C-3 in the C ring of the flavonoid skeleton. Thus, in these compounds, C-2 bears one hydrogen atom in addition to the phenolic B-ring, and C-3 has two hydrogen atoms (Grayer and Veitch, 2006). Flavanones have anti-oxidant, anti-inflammatory, and neuroprotective activity (Hernández-Aquino et al., 2017).

### Flavan-3-ols (flavanols)

The flavanols or catechins, also referred to as flavan-3-ols, are non-glycosylated compounds that are present in plants in the form of monomers (catechins). The hydroxyl group is bound to position 3 of the C ring, and has no double bonds between positions 2 and 3. Another important characteristic is the high nucleophilicity of their A-rings to HO^−^ and RO^−^ (Andersen and Markham, 2006; Panche et al., 2016). These compounds have anti-oxidant, anti-inflammatory and anti-microbial properties. The most studied flavan-3-ol monomers are (+)-catechins, (–)-epicatechin, (–)-epigallocatechin, (–)-epicatechin gallate, (–)-epigallocatechin gallate, and (+)-gallocatechin (Panche et al., 2016; Borges et al., 2017; Kuhnle, 2017).

### Anthocyanidins

Anthocyanidins have an -OH group in position 3 but also have a double bound between carbons 3 and 4 of ring C. This group is the main flavonoid responsible for cyanic colors (red, purple, and blue) in vegetables, flowers and fruits. The most common examples are cyanidin, delphinidin, pelargonidin, malvidin, and peonidin (Andersen and Markham, 2006; Azzini et al., 2017). They also have anti-oxidant and anti-microbial effects (Middleton et al., 2000; Pietta, 2000; Azzini et al., 2017; Khoo et al., 2017).

### Chalcones

The chalcones are referred to as open-chain flavonoids. Chalcones are the yellow to orange flower pigments of some plants (Andersen and Markham, 2006; Panche et al., 2016). The A and B rings of chalcones are linked by a three-carbon chain instead of a C ring, which is absent (Veitch and Grayer, 2006). Chalcones have nutritional and biological benefits owing to their anti-bacterial and anti-parasitic activities (Nowakowska, 2007; Costa et al., 2016).

### Isoflavonoids

The isoflavones are commonly known as ß-glucosides and have a B ring in position 3. These compounds have potent anti-oxidant activity. Genistein and daidzein are the main two soy isoflavones, whose main effects are the inhibition of lipid peroxidation (Lapcík et al., 1998; Yu et al., 2016). Genistein and daidzein present anti-oxidant activity in peripheral blood lymphocytes, increasing DNA protection against oxidative damage, which contributes to homeostasis in humans (Takahashi et al., 2009).

## Flavonoids and anti-oxidant properties

The imbalance between the generation of ROS and reactive nitrogen species (RNS) and their elimination is classically described as “oxidative stress,” which plays an important role in the pathophysiology of many diseases because ROS and RNS can react with lipids, proteins and DNA, inducing their oxidation and causing cell damage. The human body has several anti-oxidant systems, where anti-oxidants are understood as “any substance that retards, prevents or removes oxidative damage to target molecules” (Halliwell and Gutteridge, 2015). The most important anti-oxidant enzymes in mammalian cells are superoxide dismutase (SOD), catalase (CAT), and glutathione peroxidase (GPx) (Valko et al., 2007; Deponte, 2013; Losada-Barreiro and Bravo-Díaz, 2017). Non-enzymatic mechanisms include iron-binding proteins such as transferrin and ferritin, melatonin, and uric acid (Othman et al., 2008; Pizzino et al., 2017). These endogenous anti-oxidant systems are complementary and usually sufficient to prevent oxidative damage to the cells, but in certain conditions, such as when ROS production is excessive, the intake of exogenous anti-oxidants is convenient to reduce damage. These anti-oxidants derived from the diet are found in all vegetables and include phenols, phenolic acids, tannins, lignans, and flavonoids (Pizzino et al., 2017). The human diet contains between 50 mg and 800 mg of flavonoids per day, depending on the consumption of fruits and vegetables.

In general, the anti-oxidant activity of a flavonoid depends on three of its structural characteristics: (a) the presence of the catechol group in the B ring, (b) a Δ2 double bond and a 4-oxo group in the C ring, and (c) the hydroxyl groups on positions C-3 and C-5 (Wolfe and Liu, 2008; Kumar et al., 2013). These activities are modulated by three general mechanisms: (a) scavenging the ROS, (b) suppressing the formation of free radicals by enzymatic inhibition or chelating elements involved in the formation of them, and (c) protecting or upregulating the anti-oxidants. The ROS-scavenging activity is carried out by direct donation of hydrogen atoms, resulting in more stable and less reactive radicals.

Furthermore, some flavonoids can scavenge ${\text{O}}_{2}^{-}$ and others scavenge NO (Hanasaki et al., 1994; Vanacker et al., 1995; Prior and Cao, 2000). Anthocyanidins present the most effective scavenger function, with an activity 10–1,000 times greater than glutathione (GSH) (Cao et al., 1997). The activity of scavenging ${\text{O}}_{2}^{-}$ and NO prevents the formation of ONOO^−^, which is highly oxidizing. Moreover, flavonoids are direct chelators of ONOO^−^, as was reported for quercetin (Haenen et al., 1997; Heijnen et al., 2001; Spencer et al., 2003).

However, flavonoids that chelate Fe^2+^ or Cu^+^ can remove a causal factor in the formation of free radicals (e.g., quercetin) (de Groot and Rauen, 1998). These natural compounds stimulate the induction of anti-oxidant enzymes, such as glutathione-S transferase, UDP-glucuronosyl transferase, and NADH-quinone oxidoreductase, which are the main defense against toxic electrophilic and oxidant stress (Procházková et al., 2011). Other enzymes inhibited by flavonoids are those involved in the metabolism of arachidonic acid, such as lipoxygenase, cyclooxygenase, microsomal succinoxidase, and NADH oxidase (de Groot and Rauen, 1998). The anti-oxidant function of flavonoids includes the reduction of α-tocopherol, which represents the main anti-oxidant of membranes and low-density lipoproteins (LDL). Flavonoids can donate a hydrogen to α-tocopherol radicals and thus protect from oxidation to LDL (Hirano et al., 2001), as observed for quercetin and catechins (Zhu et al., 2000).

Although the anti-oxidant role of flavonoids is well-documented, many studies have reported their pro-oxidant activity, which seems to depend on their concentration and is directly proportional to the total number of OH groups in the molecule, especially those located in the B ring (Perron et al., 2011).

## Oxidative and anti-oxidative microenvironment in amebiasis

During ALA, *E. histolytica* is capable of inducing an important inflammatory response, which is mainly composed of neutrophils (PMNs) and macrophages. These cells create an oxidative stress environment. In the initial stages of ALA, the amebas are surrounded by neutrophils and posteriorly by macrophages. Chronic inflammation allows the formation of a granulomatous reaction. This exacerbated response can lead to extensive areas of necrosis (Tsutsumi et al., 1984). In this milieu, *E. histolytica* interacts with oxidative and non-oxidative metabolites produced by the inflammatory cells. In experimental ALA, NO concentration increases in a time dependent manner, which was found in serum samples from hamsters at different stages of the infection. In this study, the authors used histochemistry to determine the presence of the NADPH enzyme that correlated with the size and severity of the lesion. With these findings, they concluded that during the establishment of ALA, *E. histolytica* trophozoites resisted the increment of NO produced by inflammatory cells; therefore, this molecule is not sufficient to eliminate the parasite in *in vivo* studies (Pacheco-Yépez et al., 2001). The presence of NO was also evaluated in intestinal amebiasis; in these reports, a significant increase in the NO levels in patients with diarrhea was found compared with the control group. These results support that this oxidative molecule possesses a central role in the pathophysiological mechanisms underlying amebiasis (Pérez-Fuentes et al., 2000; Namiduru et al., 2011).

The relevance of NO is also related with its capacity to react with other molecules that are present in this oxidative environment, such as the superoxide anion (${\text{O}}_{2}^{-}$), which leads to the production of peroxynitrite (ONOO^−^), which has been described as one of the most potent oxidants produced in a biological system. To date, there are no reports related with peroxynitrites in amebiasis. It is necessary to evaluate the role of this molecule in the pathogenesis of amebiasis (Pacheco-Yepez et al., 2014).

In this oxidative milieu, the production of ${\text{O}}_{2}^{-}$ depends of the activity of the NADPH-oxidase, besides the SOD enzyme converts the superoxide anion to H_2_O_2_, which activates the myeloperoxidase system (MPO). This enzyme catalyzed the production of hypochlorous acid (HOCl), a highly oxidant molecule, as cytotoxic effector synthetized mainly by neutrophils. The anti-amebic activity of the MPO was described in *in vivo* experiments. The MPO caused important damage in trophozoites. These results showed that this enzyme, which was produced by inflammatory cells, is capable of protecting host tissue against *E. histolytica* (Pacheco-Yépez et al., 2011).

As was previously mentioned, trophozoites are exposed to oxidative stress (ROS and RNS such as ${\text{O}}_{2}^{-}$, H_2_O_2_, ONOO^−^, and NO). In these conditions, the ameba displays endogenous anti-oxidant enzymes to avoid the effect of these toxic molecules; therefore, *E. histolytica* can survival in this environment. A trypanothione reductase in *E. histolytica* was identified (Ondarza et al., 2005; Tamayo et al., 2005). This enzyme possesses anti-oxidant properties similar to glutathione and thioredoxin enzymatic systems (Krauth-Siegel and Comini, 2008; Krauth-Siegel and Leroux, 2012).

A 29 kDa thiol-dependent peroxiredoxin protein (Eh2CysPrx or Eh29) has been reported. The authors demonstrated that the recombinant protein presented thiol peroxidase activity due to its ability to remove H_2_O_2_. In addition, they identified the protein in the ameba cytoplasm with a molecular weight of 29 kDa (Bruchhaus et al., 1997). In contrast, other research groups reported that peroxiredoxin was localized in the membrane, and the authors demonstrated that *E. histolytica* presented a larger amount of this enzyme compared with *E. dispar*. These results showed that peroxiredoxin can be a virulence factor of *E. histolytica*, protecting trophozoites from oxidative stress (Choi et al., 2005). When the amebas were exposed for 1 h to high oxygen concentrations, the expression of *eh29* was increased 2.1-fold. The authors concluded that the enzyme is involved in the detoxification of peroxides and peroxynitrites (Akbar et al., 2004; Sen et al., 2007).

More recently, the thioredoxin system (EhTRXR/TRX), a group of anti-oxidant enzymes, has been described in *E. histolytica*. The recombinant protein is able to catalyze the reduction of NADPH or NADH and S-nitrosothiols. This enzyme exhibited NADPH dependent oxidase activity, which generates H_2_O_2_ from O_2_. This protein represents an important mechanism to regulate intracellular and extracellular levels of oxidative molecules (Arias et al., 2012). It is important to mention that these anti-oxidant systems maintain a redox balance in the parasite, allowing their survival in adverse oxidative conditions (Arias et al., 2007, 2008).

Based on these findings, we strongly suggest that flavonoids could participate as natural anti-oxidant compounds in the highly oxidative environment during ALA, favoring the resolution of liver tissue damage caused not only by the presence of the ameba and its secretion products but also by the inflammatory response characteristic of this pathology. In addition, it is important to consider the role of these natural molecules that could act directly on trophozoites. Further studies are necessary to determine the effect of flavonoids in *in vivo* models to identify their possible molecular targets in amebiasis (**Figure 5**).

## Flavonoids and their molecular targets in protozoa

Flavonoids and their therapeutic applications in human health have been extensively investigated in recent years due to their use in traditional medicine. Interestingly, these studies demonstrated a possible correlation between the chemical structure of the flavonoid and the molecular target in different cell lines (Panche et al., 2016). However, the mechanisms of action and the multiple effects of these natural compounds on the cells are not fully understood.

**Figure 2:**
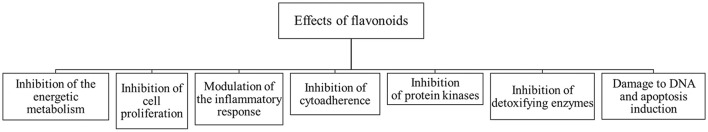
Effects of flavonoids in pathogenic protozoa.

The anti-protozoa effect of flavonoids and some of their molecular targets have been demonstrated in *Plasmodium falciparum, Trypanosoma brucei* brucei, *T. brucei* gambiense, *T. cruzi, Leishmania donovani, Cryptosporidium parvum, Toxoplasma gondii*, and *Giardia lamblia*. In *P. falciparum* the catechins acts against some identified molecular targets that include enzymes involved in the biosynthesis of fatty acids (FabG, FabZ, FabI, and enoyl-ACP reductase) (Sharma et al., 2007). In *Trypanosoma cruzi*, (–)-epicatechin has been described to affect the arginine kinase activity and NADH-oxidase activity (Paveto et al., 2004; Maya et al., 2007; Scotti et al., 2010; Dodson et al., 2011). In *Leishmania donovani*, it was reported that kaempferol promotes the inhibition of the activity of pyruvate kinase, the dihydroorotase enzyme (LdDHOase) and the cytidine deaminase, which impact the pyrimidine biosynthesis pathway, causing the death of the parasites (Scotti et al., 2015; Tiwari et al., 2016). In the case of *Toxoplasma gondii*, quercetin inhibits the synthesis of HSP90, HSP70, and HSP27, that have been described as virulence factors (Dobbin et al., 2002; Kerboeuf et al., 2008). These alterations promote reductions in the invasion to the host tissues, adhesion, proliferation and cell differentiation (Calzada et al., 1998; Mamani-Matsuda et al., 2004; Mead and McNair, 2006; Kerboeuf et al., 2008; Sen et al., 2008; Dodson et al., 2011; Jin et al., 2014; Cornelio et al., 2017) (Figure 2).

## Flavonoids with anti-amebic activity

**Figure 3:**
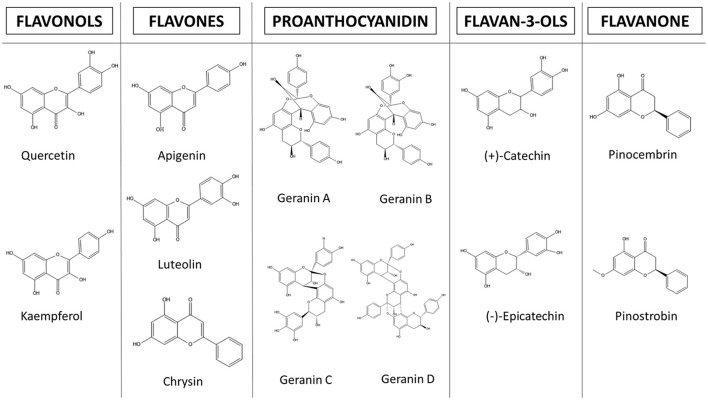
Structures of flavonoids with anti-amebic properties.

The diarrheal infections caused by *E. histolytica* represent a great problem in developing countries. They are responsible for a considerable number of morbidities and mortalities in these populations. The development of novel and effective anti-amebic compounds without side effects is necessary. Since the 1990s, medicinal plants have gained popularity as potential therapeutic alternatives. Because of this, in the last three decades, natural drugs and their products have represented approximately 50% of all treatments that have come to market (Newman and Cragg, 2016), and flavonoids display significant anti-amebic activity in *in vitro* studies (Figure 3 and Table 3).

**Table 3:** *In vitro* IC_50_ of flavonoids against *E. histolytica*.

**Flavonoid**	^*^IC_50_ (μg/ml)	**References**
(–)-epicatechin	1.9	Alanis et al., 2003; Calzada et al., 2005, 2017c
(–)-epigallocatechin	6.89	Calzada et al., 1998; Meckes et al., 1999
Kaempferol	7.93	Calzada et al., 1998, 2005; Pérez-González et al., 2017
Apigenin	10.06	Calzada et al., 1999, 2017a; Cimanga et al., 2006
Geranin B	13.6	Meckes et al., 1999
Isoquercitrin	14.7	Calzada and Alanis, 2007
Tiliroside	17.5	Calzada et al., 1999, 2017a; Calzada and Alanis, 2007
(+)-catechin	17.67	Al-Jaber et al., 2010
Luteolin	17.8	Cimanga et al., 2006
Geranin D	28.6	Calzada et al., 2001
(+)-catechin-3-*O*-α-L-rhamnopyranoside	29.67	Al-Jaber et al., 2010
Geranin C	52	Calzada et al., 2001
Quercetin	114.30	Calzada et al., 1999; Cimanga et al., 2006
Geranin A	184.7	Calzada et al., 1998; Meckes et al., 1999

* *IC_50_ values correspond to the mean concentration of the highest anti-amebic activity reported in the references*.

**Figure 4:**
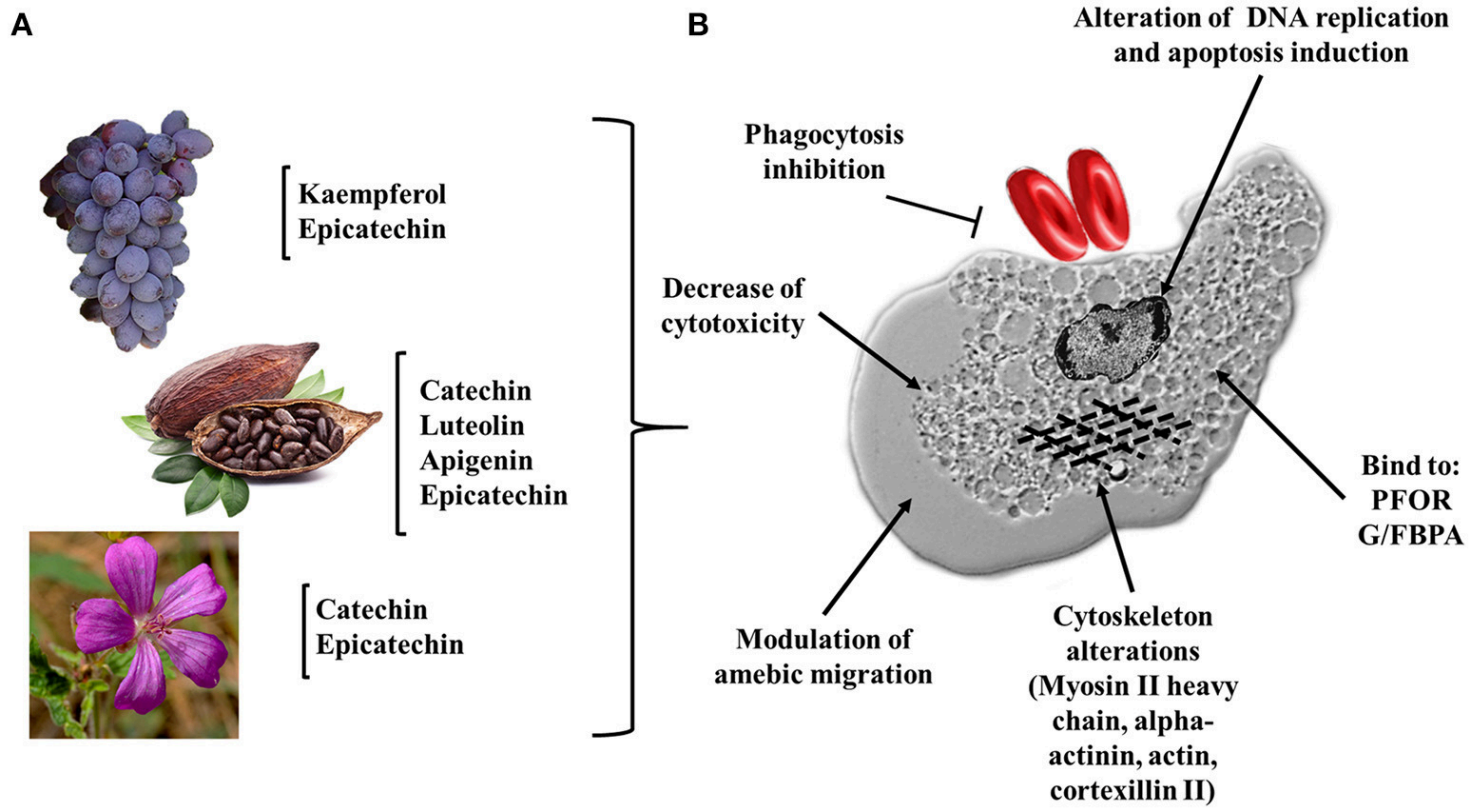
Flavonoids and their possible targets on *E. histolytica*. **(A)** Natural source of flavonoids with anti-amebic activity, fruits (grapes), seeds (cacao), and flowers (*Geranium mexicanum*). **(B)** Potential cellular targets in the ameba. Alteration of DNA replication and apoptosis induction; dysregulation of cytoplasmic proteins: HSP70, PFOR, G/FBPA, and GAPDH; inhibition of cytotoxicity, phagocytosis and alteration of the trophozoite migration (myosin II heavy chain, alpha-actinin, actin, and cortexillin II).

In recent years, flavonoids have generated great interest in the scientific community. However, there are few studies concerning their molecular mechanisms against *E. histolytica*. The most studied are kaempferol, (–)-epicatechin and tiliroside. In these studies, it has been observed that the main molecular targets correspond to cytoskeleton related proteins (myosin II heavy chain, α-actinin, and actin). The authors also demonstrated a dysregulation of glycolytic enzymes and stress oxidative proteins. They concluded that all these changes modify the pathogenic mechanism, such as adhesion, cytolysis, phagocytosis, and migration (Bolaños et al., 2014, 2015; Calzada et al., 2017a) (Figure 4 and Table 4). Below, we highlight the therapeutic effectiveness of the main flavonoids with anti-amebic activity and explain in more detail the molecular targets that could probably be affected in the ameba.

**Table 4:** Flavonoids with anti-amebic activity.

**Flavonoids**	**Targets**	**Mechanism of actions**	**References**
***IN VITRO*** **STUDIES**			
(–)-epicatechin	Nucleus	Alteration of DNA, increase in the number of glycogen deposits, reduction in the number and size of vacuoles and apoptosis induction	Soto et al., 2010
	Actin cytoskeleton and metabolic proteins (G/FBPA and GAPDH)	Alteration of migration, adhesion, phagocytosis due to modifications of alpha-actinin, myosin II heavy chain and actin proteins	Bolaños et al., 2014
Kaempferol	Inhibition of PFOR	Dysregulation of metabolism	Calzada et al., 2017b
	Actin cytoskeleton, and metabolic proteins (Aldehyde-alcohol deshydrogenase II, G/FBPA and GAPDH)	Alteration of migration, invasion and phagocytosis due to modification myosin II heavy chain and cortexillin II	Bolaños et al., 2015
Tiliroside	Inhibition of PFOR and G/FBPA	Dysregulation of glycolytic enzymes	Calzada et al., 2017a
***IN VIVO*** **STUDY**			
Resveratrol (Polyphenol)	Cell growth arrested, generation of oxidative stress, damage cell membrane lipids	Apoptosis and autophagy, decreased *in vitro* and *in vivo* liver damage	Pais-Morales et al., 2016

## *In vitro* studies and molecular targets

### Catechin

Catechin has been isolated from plants that are used in traditional medicine for the treatment of gastrointestinal disorders. Plants with anti-amebic activity that have been used as sources of catechins and their derivatives include *Helianthemum glomeratum, Osyris alba, Chiranthodendron pentadactylon, Geranium niveum, Geranium mexicanum*, and *Rubus coriifolius* (Meckes et al., 1999; Alanis et al., 2003; Al-Jaber et al., 2010; Calzada et al., 2017c).

**Figure 5:**
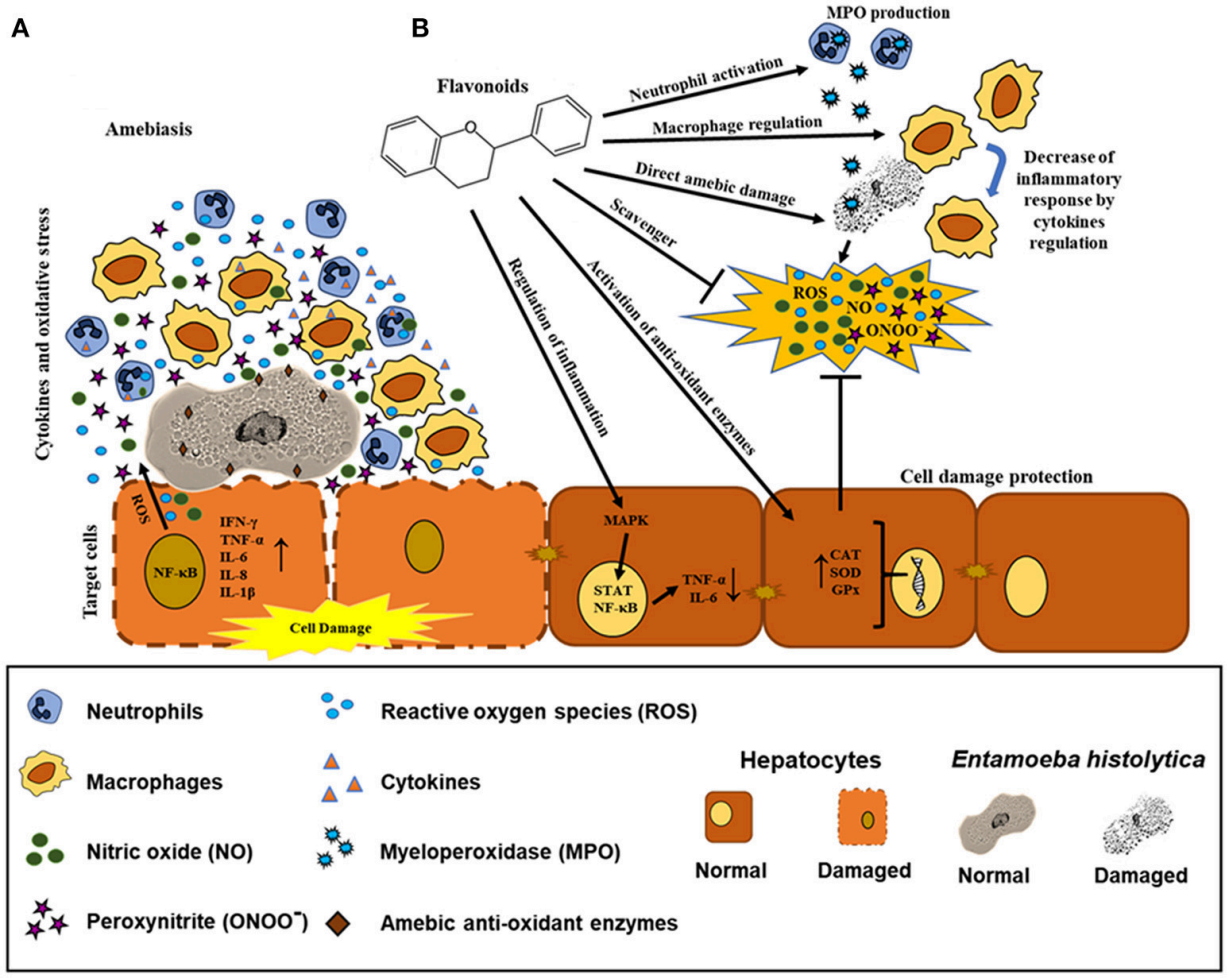
Possible effects of flavonoids in the regulation of biochemical and immunological responses against amebiasis. **(A)** Recruitment of neutrophils and macrophages by *E. histolytica* promoting the synthesis and production of pro-inflammatory cytokines and oxidative mediators. *E. histolytica* presents detoxifying enzymes. **(B)** Possible mechanisms of the flavonoids as direct scavenger of ROS, ONOO^−^, and NO, enhancer of CAT, SOD, and GPx enzymes and regulation of inflammation via STAT and NF-κB. Effect of the flavonoids in neutrophils (MPO production), macrophages (decrease of inflammatory mediators), and *E. histolytica* trophozoites (direct damage in different molecular targets).

The (–)-epicatechin isoform in *in vitro* assays has shown the best IC_50_ value of 1.9 μg/ml in inhibiting amebic growth compared with that of other catechin derivatives [e.g., (–)-epigallocatechin, (+)-catechin-3-*O*-α-L-rhamnopyranoside, and geranins A, B, C, and D] (Meckes et al., 1999; Calzada et al., 2001). The differences in the anti-amebic activity of the epicatechin derivatives can be related with the presence of hydroxy groups in the phenolic ring and galloyl moieties [e.g., (–)-epicatechin and (–)-epigallocatechin]. In addition, the presence of hydroxy groups in position 3, 4, and 5 in the B ring enhances the anti-oxidant and scavenging activities (Mizushina et al., 2005; Braicu et al., 2011) (Figure 5). The (–)-epicatechin induced morphological changes in the trophozoites at the nuclear and cytoplasmic levels, causing programmed cell death in approximately 95% of amebas (Soto et al., 2010). Moreover, (–)-epicatechin caused alterations of cytoskeleton proteins from *E. histolytica* (myosin II heavy chain, actin and alpha-actinin), affecting adhesion, cytolysis, migration, and phagocytosis (Table 4). In addition, (–)-epicatechin caused the dysregulation of enzymes that are involved in energy metabolism, such as glyceraldehyde-phosphate dehydrogenase and fructose-1,6-bisphosphate aldolase (G/FBPA). The (–)-epicatechin concentration used did not promote cytotoxicity in intestinal mammalian cells (Caco-2) (Bolaños et al., 2014). Therefore, (–)-epicatechin can be considered the most promising alternative, safe flavonoid for treatment against amebiasis. It is necessary to evaluate in more detail the molecular mechanisms involved in the specific targets and their impact on the virulence of *E. histolytica* (Figures 4, 5).

### Kaempferol

One of the first studies of the anti-amebic activity of kaempferol showed that the purified compound from the aerial parts of *Cuphea pinetorum* had an inhibitory effect on amebic culture proliferation. This work reported an IC_50_ value near 7.9 μg/ml (Calzada, 2005). Two later studies determined similar IC_50_ values against *E. histolytica*. The specific anti-amebic activity of kaempferol is independent of the plant species [*Cnidoscolus chayamansa* (Mc Vaugh) and *Annona cherimola* Miller] (Calzada et al., 2017b; Pérez-González et al., 2017). Some of the molecular mechanisms triggered by kaempferol in amebas include the dysregulation of actin, myosin II heavy chain, cortexillin II, heat shock protein 70, glyceraldehyde-phosphate dehydrogenase and G/FBPA. These results are similar to the results for (–)-epicatechin (Bolaños et al., 2014, 2015). The authors showed that at 27.7 μM, kaempferol inhibited 77.1% of ameba growth in comparison with trophozoites untreated with this flavonoid. In addition, they reported the inhibition of adhesion to Caco-2 cells in amebas co-incubated with kaempferol. (Bolaños et al., 2014, 2015; Table 4).

Kaempferol has a high affinity for pyruvate ferredoxin oxidoreductase (PFOR), an important therapeutic target in *E. histolytica* (Samarawickrema et al., 1997; Jeelani and Nozaki, 2016). By docking analysis, the authors found that the molecular interaction of kaempferol with the amebic PFOR included nine amino acid residues (Phe 665, Pro 666, Leu 667, Gly 845, Ala 846, Met 851, Tyr 853, Trp 864, and Asn, 866) compared with the four different binding sites of MTZ (Phe 174, His 178, Lys 435, Phe 453, and Tyr 455) (Calzada et al., 2017b).

Kaempferol derivatives also exhibit considerable anti-amebic activity. In particular, tiliroside showed an IC_50_ of 7.45 μg/ml against *E. histolytica* trophozoites. In a similar manner to kaempferol, tiliroside can interact directly with PFOR and G/FBPA according to docking studies, showing an inhibition constant (KI) of 53.57 and 55.5 μM, respectively. These data are comparable with the values of the KI for MTZ (47.64 and 44.01 μM, respectively) (Calzada and Alanis, 2007; Calzada et al., 2017a) (Figure 4 and Table 4). It is important to remark that the differences in IC values and the molecular mechanisms of action between kaempferol and its derivatives may be due to the presence of glucosyl moieties on position C3 of the principal structure or the number of hydroxyl groups in ring B, which probably limits the correct activity of tiliroside (Cimanga et al., 2006; Singh et al., 2009) (Table 3).

Based on previous reports, kaempferol, (–)-epicatechin, and (–)-epigallocatechin present different IC_50_ for anti-amebic activity and presented the best IC_50_ values that showed good activity against *E. histolytica*. These natural compounds reduced the amebic viability by 50% (1.9, 6.89, and 7.93 μg/ml respectively). Due to the low IC_50_, these flavonoids could be considered good candidates to evaluate their *in vivo* activity. Other important advantages of the use of flavonoids are their anti-oxidant and anti-inflammatory activity, which could participate in the resolution of amebiasis.

### Quercetin

This flavonoid showed a slight anti-amebic effect *in vitro* (IC_50_ 114.3 μg/ml) compared with that of *G. lamblia* (IC_50_ 26.6 μg/ml). However, isoquercitin, a quercetin derivative, inhibited the viability and growth of *E. histolytica* trophozoites after 48 h of incubation (IC_50_ 14.7 μg/ml) (Calzada and Alanis, 2007). Although there are no reports of a specific target of isoquercetin in the ameba, it is imperative to mention that this flavonoid is metabolized in the bowel and the liver (Valentova et al., 2014); therefore, its administration in *in vivo* models could diminish the tissue damage induced by *E. histolytica*.

### Other polyphenol compounds with anti-amebic effects

Other flavonoids that have anti-amebic activity are luteolin and apigenin. These compounds, isolated from the total extract of *Morinda morindoides*, have shown IC_50_ values of 17.8 and 10 μg/ml, respectively, against the ameba. The authors suggested that glycosylation on the C-7 position decreased the anti-amebic activity of these flavonoids (Cimanga et al., 2006) (Table 3). Nevertheless, it is important to mention that the anti-amebic mechanisms of these flavonoids and their real potential activities have not been determined. Other compounds with anti-amebic effects are the chalcones. Leeza Zaidi et al. (2015) synthetized modified chalcones (N-substituted ethanamine). *In vitro* assays showed that their derivatives displayed a better anti-amebic activity than the MTZ reference drug (Leeza Zaidi et al., 2015).

## *In vivo* studies

The flavonoids have shown extensive benefits and no side effects in *in vivo* models of different types of cancer, liver cirrhosis, neurodegenerative diseases and metabolic disorders (e.g., obesity and diabetes) (Goto et al., 2012; Nabavi et al., 2015; Panche et al., 2016; Hernández-Aquino et al., 2017).

In contrast, there are few reports of flavonoids in parasitic diseases using *in vivo* models. The efficacy of intragastric treatment with (–)-epicatechin at 0.072 μmol/kg was demonstrated in CD-1 mice infected with *G. lamblia* trophozoites (1 × 10^6^). The (–)-epicatechin administered 6 days post-infection decreased the number of parasites in the small intestine. Moreover, this natural compound displayed a higher activity than that of MTZ and emetine (Barbosa et al., 2007). The epigallocatechin was effective in BALB/c mice infected with *Leishmania amazonensis*. The results showed that the animals treated orally with 30 mg/kg/day of epigallocatechin for 5 days presented a reduction in the size of the ear lesion and a low parasite burden compared with that of the control group (Inacio et al., 2013).

It is important to mention that, until now, there have been no studies of amebiasis using flavonoids in *in vivo* models. Nevertheless, a polyphenol, resveratrol, has presented anti-amebic properties both *in vitro* and *in vivo*. *In vitro* incubation with resveratrol (72 μM) for 48 h induced cell growth arrest, production of ROS, damage to membrane lipids, increased intracellular Ca^2+^, calpain activation, and decreased superoxide dismutase activity, leading to apoptosis in *E. histolytica*. Moreover, in hamsters with ALA, the pre-treatment for 2 or 10 days with 30 mg/kg of resveratrol by oral gavage prevented liver damage by the trophozoites, whereas non-pretreated animals developed extensive ALA (80% of the total liver). Histopathological analysis showed that resveratrol-treated hamsters presented a healthy liver parenchyma with retraction of the granulomatous reaction, whereas the untreated animals displayed liver necrosis. Based on these results, the authors suggested that resveratrol could be used as alternative treatments for amebiasis (Pais-Morales et al., 2016). Although resveratrol is a polyphenol, it is necessary to perform *in vivo* studies to demonstrate the potential anti-amebic effect of flavonoids before their use as an alternative or complementary treatment.

## Closing remarks

In recent years, the use of natural compounds against infectious diseases caused by protozoan parasites has gained popularity among pharmaceutical corporations (Newman and Cragg, 2016). In 2015, the Nobel Prize in Medicine was awarded to Professor Youyou Tu for her valuable contributions to the discovery of artemisinin as a natural malaria treatment (Su and Miller, 2015). These advances have been a consequence of the various reports on drug resistance in microorganisms as well as the toxicity and side effects of many drugs on humans. The field of amebiasis is no exception. Promising advances have been made using different secondary metabolites from plant extracts (Procházková et al., 2011; Herrera-Martínez et al., 2016; Bashyal et al., 2017). The use of plants rich in flavonoids (grapes, cacao and flowers such as *Geranium mexicanum*) have been shown to exert anti-amebic activity, having cytoskeletal proteins and enzymes related to the glycolytic metabolism of *E. histolytica* as molecular targets and leading to alterations in DNA replication. These changes suggest that the ameba lost their virulence factors (Bolaños et al., 2014, 2015) (Figure 4). It is well known that during the establishment of amebiasis in the intestine and in the liver, the inflammatory reaction can promote the participation of pro-inflammatory cytokines. In this milieu, there are a highly oxidative stresses, constituted by NO, peroxynitrites (ONOO^−^), ROS, and hypochlorous acid. All these oxidant metabolites caused tissue damage. Flavonoids can regulate inflammation-activating anti-oxidants enzymes (CAT, SOD, and GPx). Additionally, flavonoids participate as scavengers that can remove these free radicals. We cannot discard that flavonoids act directly with neutrophils and macrophages to kill amebas (Figures 5A,B).

Considering all the benefits and probable therapeutic targets of flavonoids in the treatment of *E. histolytica* infection, it is important to investigate these natural compounds in basic research studies that will establish the doses, administration routes, bioavailability, and metabolic biotransformation (glycosidation, glucuronidation, sulfation, and O-methylation) as well as effects on the tissue microenvironment of these natural compounds in *in vivo* models of amebiasis. Considering all of these remarks, flavonoids can be considered good alternatives for the effective treatment of amebiasis.

## Author contributions

Authors that contributed to writing the manuscript: MS (introduction and closing remarks), JP-Y and RC-R (flavonoids structure and classification), RJ-L (flavonoids and anti-oxidant properties), NF-H and LC-J (flavonoids and their molecular targets in protozoa), MM-C and PG-T (flavonoids with anti-amebic activity; *in vitro* and *in vivo* studies and molecular targets). MS organize and revise the manuscript. All the authors prepared and edited the figures and tables. All authors contributed to manuscript revision, read and approved the submitted version.

### Conflict of interest statement

The authors declare that the research was conducted in the absence of any commercial or financial relationships that could be construed as a potential conflict of interest.
